# A modulation-doped heterostructure-based terahertz photoconductive antenna emitter with recessed metal contacts

**DOI:** 10.1038/s41598-020-76413-7

**Published:** 2020-11-16

**Authors:** Jessica Afalla, Alexander De Los Reyes, Neil Irvin Cabello, Victor DC Andres Vistro, Maria Angela Faustino, John Paul Ferrolino, Elizabeth Ann Prieto, Hannah Bardolaza, Gerald Angelo R. Catindig, Karl Cedric Gonzales, Valynn Katrine Mag-usara, Hideaki Kitahara, Armando S. Somintac, Arnel A. Salvador, Masahiko Tani, Elmer S. Estacio

**Affiliations:** 1 Graduate School of Pure and Applied Sciences, University of Tsukuba, Tsukuba, 305-8573 Japan; 2 Research Center for Development of Far Infrared Region, University of Fukui, Fukui, 910-8507 Japan; 3 National Institute of Physics, University of the Philippines Diliman, 1101 Quezon City, Philippines; 4 Material Science and Engineering Program, University of the Philippines Diliman, 1101 Quezon City, Philippines

**Keywords:** Terahertz optics, Ultrafast photonics, Photonic devices

## Abstract

We present the implementation of an efficient terahertz (THz) photoconductive antenna (PCA) emitter design that utilizes high mobility carriers in the two-dimensional electron gas (2DEG) of a modulation-doped heterostructure (MDH). The PCA design is fabricated with recessed metal electrodes in direct contact with the 2DEG region of the MDH. We compare the performance of the MDH PCA having recessed contacts with a PCA fabricated on bulk semi-insulating GaAs, on low temperature-grown GaAs, and a MDH PCA with the contacts fabricated on the surface. By recessing the contacts, the applied bias can effectively accelerate the high-mobility carriers within the 2DEG, which increases the THz power emission by at least an order of magnitude compared to those with conventional structures. The dynamic range (62 dB) and bandwidth characteristics (3.2 THz) in the power spectrum are shown to be comparable with the reference samples. Drude-Lorentz simulations corroborate the results that the higher-mobility carriers in the MDH, increase the THz emission. The saturation characteristics were also measured via optical fluence dependence, revealing a lower saturation value compared to the reference samples. The high THz conversion efficiency of the MDH-PCA with recessed contacts at low optical power makes it an attractive candidate for THz-time domain spectroscopy systems powered by low power fiber lasers.

## Introduction

The terahertz (THz) band refers to the 100 GHz–30 THz frequencies of the electromagnetic spectrum^1^. The applications of THz radiation in numerous fields, such as energy^2^, medicine^3^, manufacturing^4^, imaging^5^ and arts^6^, have driven the demand for more intense THz sources. One of the most popularly-used broadband THz sources for spectroscopic applications is the photoconductive antenna (PCA)^7^. It is typically composed of a semiconductor substrate material having two surface metal electrodes, with the electrodes separated by micrometer scale**,** forming the photoconductive gap. In its typical operation, the gap of the PCA THz emitter is biased with either a dc or ac voltage and illuminated with ultrafast laser pulses. Upon illumination, electron–hole pairs are photogenerated in the semiconductor substrate. As the electrons and holes drift in opposite directions towards the biased metal electrodes, they form a transient current, which emits THz radiation via the metal contacts functioning as an antenna^8^. The performance of the PCA is dictated by the characteristics of the photoconductive substrate, such as carrier lifetime, band gap, breakdown voltage, resistivity and electron mobility^8^. Low temperature-grown gallium arsenide (LT-GaAs) is among the most commonly used PCA substrate material due to its short carrier lifetime and its high breakdown-voltage characteristics, despite LT-GaAs having lower electron mobility compared to crystalline GaAs^9^. Meanwhile, semi-insulating (SI) GaAs PCAs have also been commercially utilized as THz emitters due instead to their high mobilities^10^. Following this approach, a more intense THz emission can be obtained from a PCA emitter that utilizes a GaAs-based, high electron mobility heterostructure; an example of which is the aluminum gallium arsenide (AlGaAs)/GaAs modulation-doped heterostructure (MDH).

**Figure 1:**
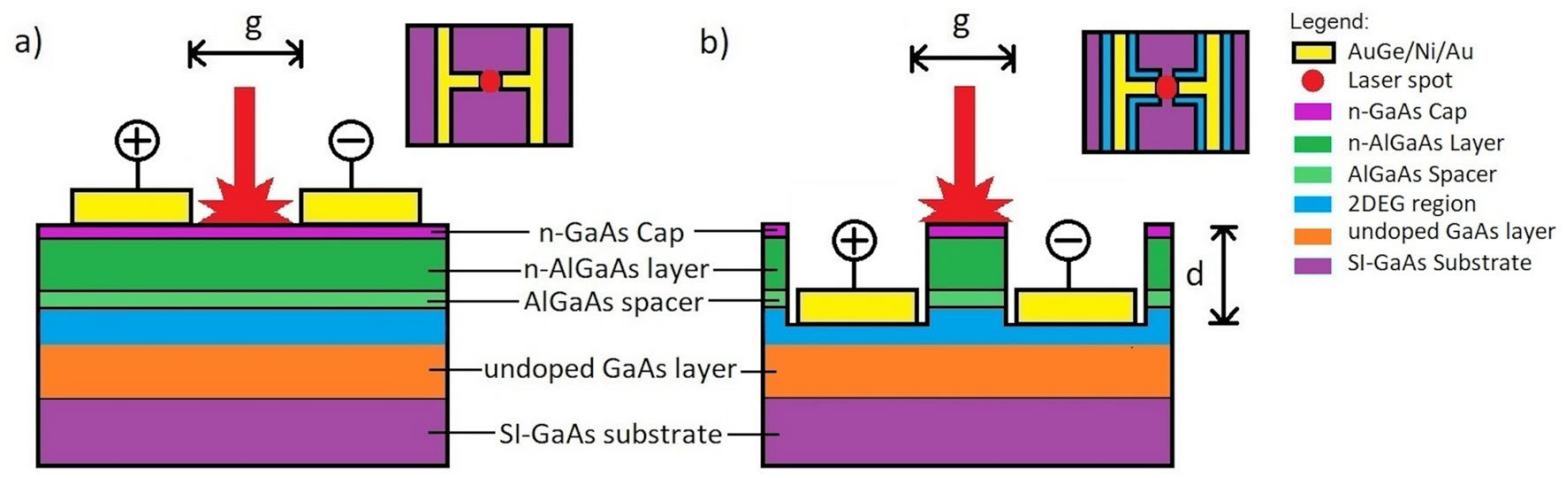
Schematic diagrams of the cross section of the MDH PCAs when the metal contacts are (**a**) at the surface of the MDH and (**b**) recessed. $g$ denotes the gap width, and $d$ denotes the etch depth. The inset figures show the top view of each of the MDH PCA. The diagrams are not to scale.

In a conventional AlGaAs/GaAs MDH, n-doped AlGaAs is separated from undoped GaAs by a thin spacer layer (Fig. 1a). Due to the alignment of the Fermi levels, a triangular well is formed in the conduction band. The confined carriers in this triangular well form a two-dimensional electron gas (2DEG) region, where the electrons have higher mobility and lower scattering from ionized impurities, compared to electrons in the bulk GaAs region^11^. Owing to the enhanced carrier mobility, the MDH is conventionally used for high-speed devices; particularly as a modulation-doped field effect transistor (MODFET), also referred to as “high electron mobility transistor (HEMT)”, as well as applications in spectroscopy^12^**,** and optoelectronics^13^. The MDH utilized as a HEMT has been previously shown by Dyakonov et al. to function in the THz range as a detector, mixer, and multiplier owing to the utilization of the 2DEG^14–16^. While this has sparked interest in MDH-based materials and devices for THz applications, previous works have delved on utilizing the MDH for THz detection^15–19^, rather than generation.

Previous works have shown that the application of an external magnetic field enhances the THz emission of several semiconductors^20,21^. We have previously observed in a bare MDH that the polarity of the applied magnetic field parallel to the surface and normal to the reflection plane dictated the THz enhancement factor^22^, and demonstrated via temperature-dependent THz-time domain spectroscopy (THz-TDS) that the high-field region in the 2DEG is responsible for the THz emission in a MDH^23^. This effect was most pronounced in the AlGaAs/GaAs MDH and highest when the external magnetic field was applied parallel to the heterojunction of the MDH. Doing so made the carriers in the MDH mimic the motion of the carriers of a PCA emitter under normal, biased operation^24^.

In this paper, we report on the characteristics of the AlGaAs/GaAs MDH utilized as a PCA with recessed metal contacts, a design we have previously proposed^24^. The MDH PCA emitter, along with standard SI-GaAs and LT-GaAs PCAs, were fabricated by standard lithography techniques and were tested via THz-TDS measurements to understand and compare each of the devices’ performance. We show that by exploiting the transport of high-mobility electrons along the 2DEG region, the MDH PCA with recessed contacts shows THz emission amplitude increased by a factor of 7 over that of a SI-GaAs PCA**,** and roughly by a factor of 1.5 over that of a LT-GaAs PCA. To analyze how the enhanced mobility and reduced scattering would affect the devices, the THz emission characteristics of the PCA devices using the different substrates, namely SI-GaAs, LT-GaAs, unrecessed MDH and recessed MDH, were simulated using the Drude-Lorentz model. With its strong THz emission and compact dimensions, the recessed MDH PCA can help pave the way to more efficient, compact, and turn-key THz spectroscopy solutions.

## Photoconductive antenna design and simulation

Figure 1 shows the cross-section of an AlGaAs/GaAs MDH PCA with surface contacts (Fig. 1a) and recessed contacts (Fig. 1b). The recessed features had an etch depth of $d$ = 187 nm. The PCA pattern used for both was a dipole antenna with a gap of $g$ = 5 µm. The recessed features could be achieved by selectively etching the layers prior to the deposition of the metal contacts. The proximity of the metal contacts to the 2DEG provides easier access for the electric bias to utilize the 2DEG region of the AlGaAs/GaAs MDH, resulting in a stronger THz wave emission. Epitaxial growth, lithography and fabrication are discussed in more detail in the Methods.

The mechanism behind the experimental results are supported by numerical simulations of the THz emission using the one-dimensional Drude-Lorentz Model^25,26^. The one-dimensional Drude-Lorentz Model is a simple, yet accurate model of the generation of THz electromagnetic radiation^25,26^. As a femtosecond optical pulse is made incident onto the photoconductive gap, the photogenerated electron–hole pairs are swept by the applied electrical bias. The transient photocurrent density $j$ is given by

1 $$j = en_{f} v_{h} - env_{e}$$

where $e$ is the electron charge, $n_{f}$ is the free carrier density, $v_{h}$ and $v_{e}$ are the average hole and electron velocities, respectively. The time-dependence of the free carrier density is given by:

2 $$\frac{{dn_{f} }}{dt} = - \frac{{n_{f} }}{{t_{c} }} + G\left( t \right)$$

where $\tau_{c}$ is the carrier capture time and $G\left( t \right)$ is the carrier generation rate of the form $n_{0} exp\left( { - t^{2} /p^{2} } \right)$ by optical excitation. The acceleration of holes and electrons is given by:

3 $$\frac{{dv_{h,e} }}{dt} = - \frac{{v_{h,e} }}{{\tau_{s} }} + \frac{{q_{h,e} }}{{m_{h,e}^{*} }}E_{mol}$$

where $v_{h,e}$ is the average velocity, $q_{h,e}$ is the charge, $m_{h}^{*} = 0.34m_{e,0}$, $m_{e}^{*} = 0.067m_{e,0}$, $\tau_{s}$ is the momentum relaxation time given by the Drude relation $\tau_{s} = \mu_{i} m_{i}^{*} /q_{i}$ and $E_{mol}$ is the local electric field given by:

4 $$E_{mol} = E_{bias} - \frac{{P_{sc} }}{\eta \varepsilon }$$

where $P_{sc}$ is the space-charge polarization created by the carrier separating due to the applied field, $\varepsilon$ is the dielectric constant of the material, and $\eta$ is the geometrical factor of the antenna. For this work, we use $\varepsilon = 12.9\varepsilon_{0}$, which is the dielectric constant of GaAs^27^.

The time dependence of the space-charge polarization is,

5 $$\frac{{dP_{sc} }}{dt} = - \frac{{P_{sc} }}{{\tau_{r} }} + j\left( t \right)$$

where $\tau_{r}$ is the recombination lifetime. Taking the time derivative of Eq. (3), then inserting Eq. (4), the second time-derivative of the velocity $v$ is given by

6 $$\frac{{d^{2} v_{h,e} \left( t \right)}}{{dt^{2} }} = - \frac{1}{{\tau_{s} }}\frac{{dv_{h,e} }}{dt} + \frac{{q_{h,e} }}{{m_{h,e}^{*} \eta \varepsilon }}\frac{{P_{sc} }}{{\tau_{r} }} - \frac{{n_{f} e^{2} v_{h,e} }}{{m_{h,e}^{*} \eta \varepsilon }}$$

Solving both Eqs. (5) and (6) and using Eqs. (1) and (2) will give the photocurrent density $j$. At far-field, the THz electric field $E_{THz} \left( t \right)$ is proportional to the time-derivative of the photocurrent density

7 $$E_{THz} \left( t \right)\sim \frac{dj}{{dt}}$$

The THz wave radiated from the emitter PCA is assumed to reach the PCA detector without any losses. The probe beam generates electron–hole pairs, and the THz electric field incident on the detector PCA sweeps the photocarriers. The current density at the PCA detector is given by^28^:

8 $$j\left( t \right) = \mathop \smallint \limits_{ - \infty }^{t} \sigma_{s} \left( {t - t^{\prime}} \right)E_{THz} \left( {t^{\prime}} \right)dt^{\prime}$$

where $\sigma_{s} \left( t \right)$ is the transient surface conductivity of the photoconductive substrate of the detector. The transient surface conductivity of the detector was modelled using a LT-GaAs substrate with $\tau_{c,det}$ = 0.15 ps, $\tau_{s,det}$ = 40 fs. These detector values best replicate the frequency response of the experimental data and are kept as constant PCA detector parameters when simulating the SI-GaAs, LT-GaAs, MDH (Top) and MDH (Recessed) PCA emitters.

The simulation requires physical parameters of the semiconductor substrates, specifically, carrier density $n_{f}$, capture time $\tau_{c}$, scattering time $\tau_{s}$, and recombination time $\tau_{r}$. These parameters have been well-documented for SI-GaAs, while the values for LT-GaAs would depend on the growth temperature. For the MDH samples, Hall mobility measurements were performed to measure the actual carrier density and mobility values. Van der Pauw configuration was utilized by applying indium contacts on top of the MDH and the magnetic field was supplied using a 3 T Lakeshore magnet.

## Results and discussion

**Figure 2:**
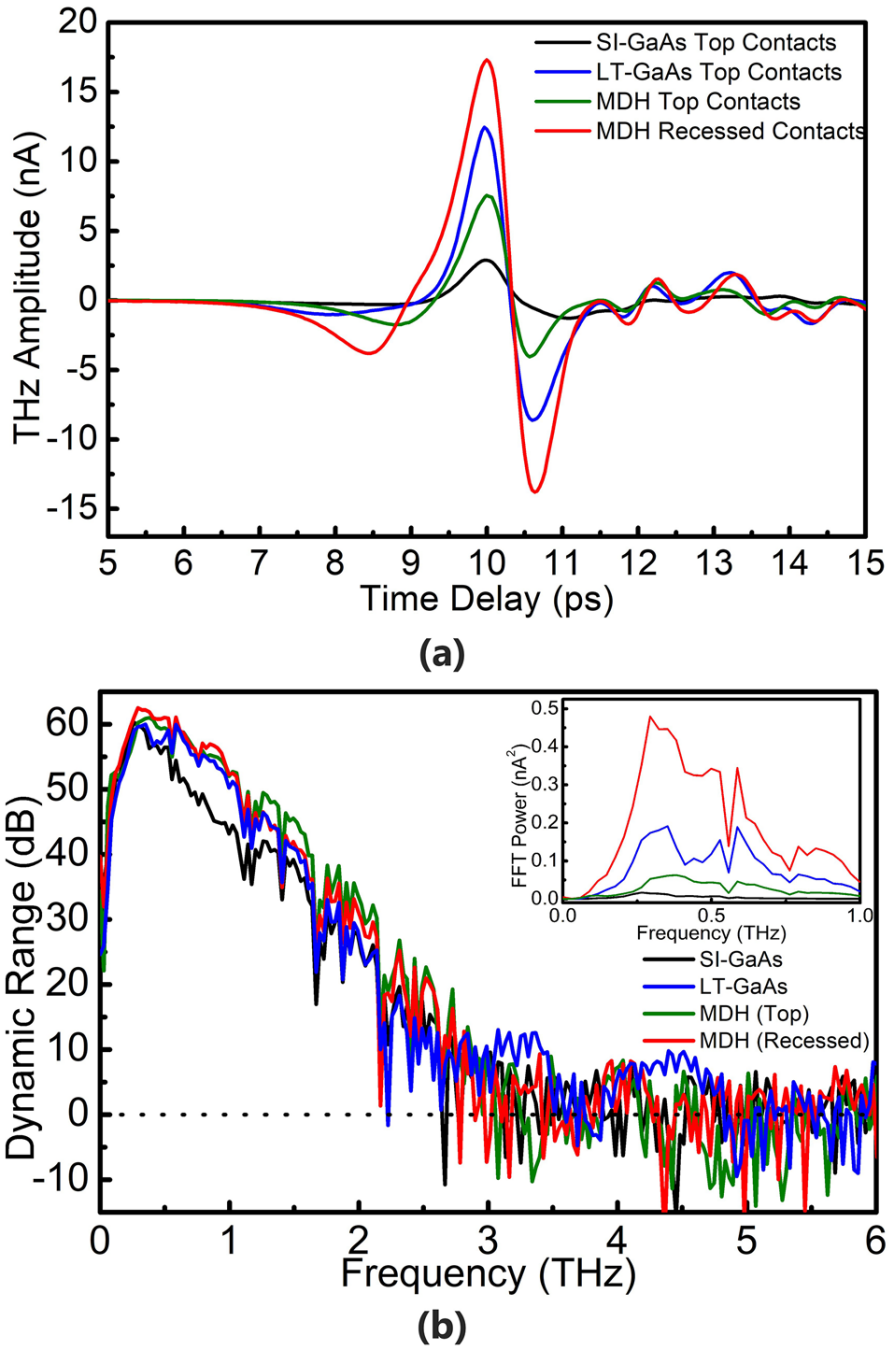
(**a**) Measured THz-TDS waveforms and (**b**) the corresponding normalized dynamic range spectra from the photoconductive antennas. Each plot was given a corresponding y-offset in order to align the noise floor average to the y = 0 dB line. The inset in (**b**) is a plot of the frequency power spectra in linear scale.

Identical dipole-type patterns (gap width $g$ = 5 μm) were fabricated on the surfaces of a SI-GaAs (100) substrate, a LT-GaAs (growth temperature 270 °C) substrate, and a MDH substrate; and on a separately prepared piece of the same MDH sample, an antenna was fabricated with the electrical contacts recessed, as described earlier. From here onwards, we refer to the fabricated PCAs as “SI-GaAs”, “LT-GaAs”, “MDH (Top)” and “MDH (Recessed)”. The PCAs were biased at a frequency of 20 kHz, and peak-to-peak voltage amplitude of 32 V. The powers of the pump beam and probe beam were both maintained at 9.5 mW, unless otherwise stated. Figure 2a shows the THz time domain emission spectra from the fabricated PCAs. The generated THz waves were detected using a commercial LT-GaAs dipole-type PCA with a 3.4 µm gap. Among the four antennas, the highest THz peak-to-peak amplitude was observed from the MDH (Recessed) PCA, followed by LT-GaAs, MDH (Top) and SI-GaAs PCAs. Between the two MDH PCAs, we find that by recessing the contacts, the bias is able to access the 2DEG region more effectively, and an increase in the drift carrier transport of carriers in the 2DEG resulted in the generation of higher THz emission.

The dynamic range of the PCAs as a function of THz frequency are shown in Fig. 2b, where each plot was given an appropriate y-offset such that the noise floor average coincides with the y = 0 dB line (dotted line). The inset shows the THz power spectra plotted in linear scale, to provide the reader with a visual context of the spectral difference in THz emission among the devices. The maximum dynamic range for all PCAs are ~ 60 dB. However, between 0.4 THz and 1.5 THz, the dynamic range of the MDH PCAs and LT-GaAs PCAs are higher, by around 10 dB at most, compared to the SI-GaAs PCA; and at frequencies higher than 1.5 THz, the MDH PCAs have a slightly higher dynamic range (~ 5 dB) than the LT-GaAs PCA. The increased density of high velocity carriers participating in the THz emission process^29^ increases the higher frequency components of the spectra. The performance characteristics are detailed in Table 1.

**Table 1:** PCA performance under 32V_pp_, 20 kHz bias and optical pump fluence 3.85 mJ/cm^2^.

PCA	THz peak to peak amplitude (nA)	Emission bandwidth (THz)	Maximum dynamic range (dB)	Integrated THz power (nA^2^·THz)
SI-GaAs	4.04	3.2	60	4.5 × 10^–3^
LT-GaAs	20.92	3.2	60	8.51 × 10^–2^
MDH (Top)	11.37	3.2	61	2.85 × 10^–2^
MDH (Recessed)	30.88	3.2	62	0.202

**Figure 3:**
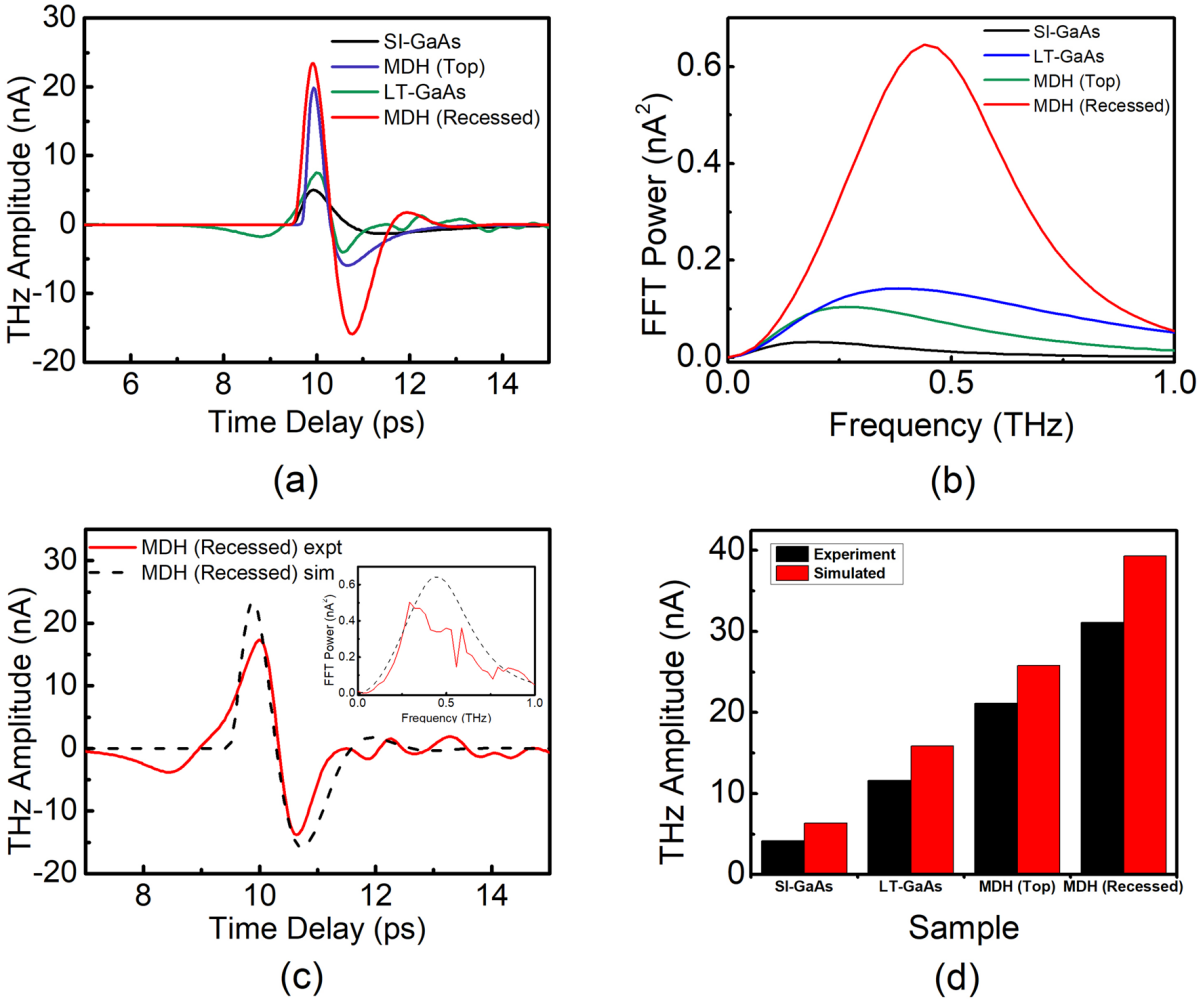
(**a**) Simulated THz-TDS waveform and (**b**) simulated FFT spectra for the samples (**c**) Representative TDS plot superimposing experimental and simulated results for MDH (Recessed) with inset as FFT spectra (**d**) Comparison between experimental and simulated peak-to-peak THz amplitude for SI-GaAs, LT-GaAs, MDH (Top) and MDH (Recessed).

The THz waveforms and FFT spectra from photoconductive antenna simulation using the one-dimensional Drude-Lorentz model are shown in Fig. 3. The parameters used for the carrier density $n_{f}$, capture time $\tau_{c}$, scattering time $\tau_{s}$ and recombination time $\tau_{r}$ used in the simulation are detailed in Table 2. The scattering time was deduced from the experimentally-obtained mobility values using the Drude relation $\tau_{s} = \mu_{i} m_{i}^{*} /q_{i}$(or vice-versa when the scattering time is known, such as for SI-GaAs and LT-GaAs). The SI-GaAs has a carrier capture time in the order of hundreds of picoseconds, a relatively high scattering time, and high mobility of > 5000 cm^2^/(V·s)^30–32^. The LT-GaAs used in this work was grown at $T_{s}$ = 270 °C and the presence of defects leads to picosecond carrier lifetime, a low scattering time and a low mobility of < 1000 cm^2^/(V·s)^9,33^. The time-scales of the MDH samples were estimated from literature values based on capacitive measurements or time-resolved measurements^34,35^. For the MDH (Top) structures, the carrier density and mobility were chosen close to actual Hall measurement values while the mobility of the MDH (Recessed) sample was scaled accordingly assuming that the radiated terahertz electric field $E_{THz}$ is directly proportional to the mobility $\mu$. The effective mobility of the carriers contributing to the source current for THz emission is improved by the direct contact of the metal to the 2DEG region.

**Table 2:** Numerical values used for the one-dimensional Drude-Lorentz PCA simulation.

PCA	Carrier density, $n_{i}$	Trapping time constant, $\tau_{c}$	Scattering time constant, $\tau_{s}$	Recombination time constant, $\tau_{r}$	Electron mobility, $\mu$
SI-GaAs	2.00 × 10^16^ cm^−3^^36^	100 ps^30^	250 fs^31^	1 ns^32^	6650 cm^2^ V^−1^ s^−1a^
LT-GaAs	1.00 × 10^18^ cm^−3^^33^	1 ps^37^	20 fs^38^	100 ps^37^	500 cm^2^ V^−1^ s^−1a^
MDH (Top)	1.00 × 10^17^ cm^−3b^	1 µs^34^	100 fs^a^	1 µs^35^	2660 cm^2^ V^−1^ s^−1b^
MDH (Recessed)	1.00 × 10^17^ cm^−3b^	1 µs^34^	250 fs^c^	1 µs^35^	6650 cm^2^ V^−1^ s^−1c^

^a^From Drude relation.

^b^From Hall measurements.

^c^Fitting parameter.

The resulting trends in the simulation are in good agreement with the experimentally measured THz radiation for both the time domain (Figs. 2a, 3a) and the frequency spectra (Figs. 2b inset, 3b). This includes the increased amplitude of higher THz frequency components with the electron mobility of carriers participating in the THz generation process. A representative comparison showing the experimental data and simulation for the MDH (Recessed) is shown in Fig. 3c. A deviation between the time-domain waveforms and FFT spectra of the experimental and simulation results is explained by the deformation or renormalization of the THz waveform due to the antenna response, the frequency-dependent focusing characteristics of the THz optics, and the water vapor absorptions, all of which have been ignored in the simulation.

A comparison between the data and the simulation is presented in a bar graph in Fig. 3d. The differences in THz emission amplitude between the data and the simulation implies that the etched distance from the surface leaves room for optimization. Nonetheless, the good agreement between the simulation results and the experimental data shows how the MDH-PCA design effectively utilizes the high-mobility 2DEG region in improving THz yield.

**Figure 4:**
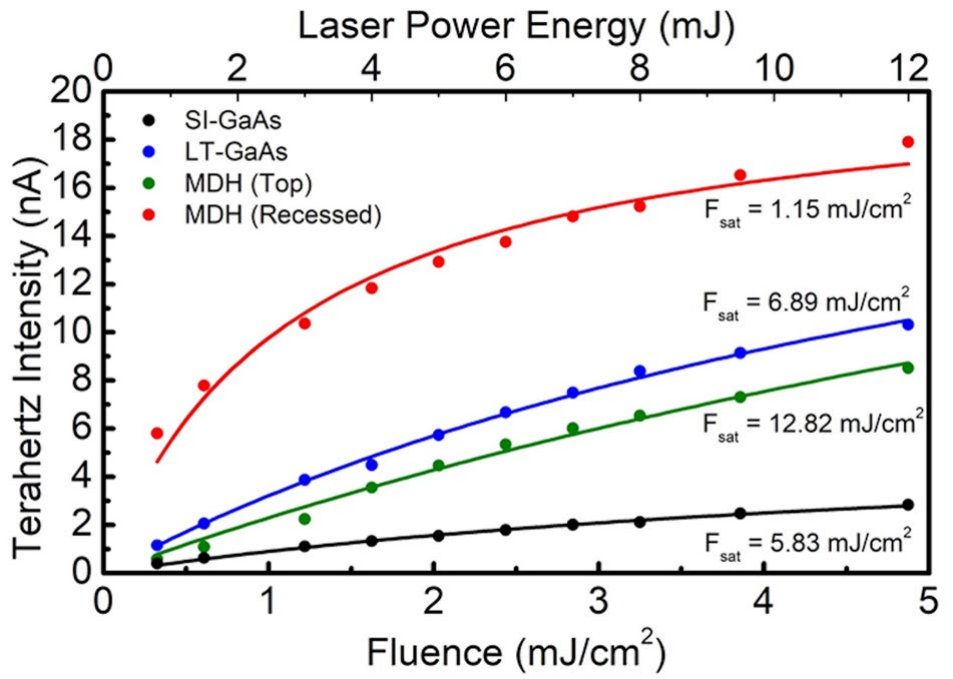
Fluence dependence of the THz peak-to-peak value of the different fabricated PCAs. The solid lines are the saturation curve fitting.

The dependence of the THz emission amplitude to the optical fluence was obtained (Fig. 4) by varying the laser power incident on the THz emitter PCAs. At any given pump fluence, the SI-GaAs PCA emits the lowest THz emission amplitude, followed by the MDH (Top) PCA, and lastly, the LT-GaAs PCA. The MDH (Recessed) PCA has the highest THz emission amplitude. Even at the lowest fluence value (< 0.5 mJ/cm^2^), the THz emission from the MDH (Recessed) PCA was 5 times higher than the THz emission of the LT-GaAs PCA. The saturation fluence $F_{sat}$ can be calculated from the fits to the equation, $E_{THz} \left( F \right) \approx A\left( {F/F_{sat} } \right)/\left( {F + F_{sat} } \right)$, where $A$ is the amplitude of the radiated field, and $F$ is the incident beam fluence. The saturation fluence values are 5.83 mJ/cm^2^ and 6.89 mJ/cm^2^ for the SI-GaAs and LT-GaAs PCAs, respectively. For the MDH (Top) PCA, the $F_{sat}$ is 12.82 mJ/cm^2^. When the contacts are recessed**,** however, the value for $F_{sat}$ significantly reduces to 1.15 mJ/cm^2^. The saturation of the emitted THz radiation from PCAs with optical fluence, in general, is attributed to the screening effect that arises from the high photocarrier density^39–41^, and the saturation fluence is inversely proportional to the carrier mobility^40^. With recessed contacts, the applied bias becomes more efficient as it directly accesses the high mobility region; in contrast to when it is applied from the surface. This improved efficiency in the bias conditions outweigh the corresponding detrimental effects of screening.

As a point of reference, LT-GaAs is the most common commercially-available photoconductive material used for emitters because of the ultrashort carrier lifetime due to the high concentration of defects^9,42^. We find that compared to LT-GaAs, the MDH (Recessed) PCA emits a higher THz peak to peak amplitude and has a greater maximum dynamic range, even as they emit at the same THz spectral bandwidth. While LT-GaAs does have a higher saturation fluence at any given moderate fluence value, the efficiency of the MDH (Recessed) PCA is consistently higher in the < 5 mJ/cm^2^ fluence range. The MDH (Recessed) PCA would be a good candidate for low laser power applications because of its high THz emission yield. When building THz-TDS spectrometers driven by compact low power fiber lasers, the efficiency of optical-to-THz power is crucial.

In summary, the previously-proposed PCA design was successfully implemented using an n-AlGaAs/GaAs MDH. The MDH was etched to recess the metal for direct contact with the 2DEG region of the MDH. As corroborated by Drude-Lorentz simulation, the influence of the high mobility carriers in the 2DEG was shown to drive the increase in THz emission. The MDH recessed contacts have the largest THz peak-to-peak emission and THz power, as compared to the LT-GaAs and MDH PCAs with top contacts; even as their dynamic and spectral ranges are comparable. The high THz emission and low saturation fluence of the MDH recessed contacts offer a feasible solution to THz-TDS systems that are designed to be powered by low power fiber lasers.

## Methods

**Figure 5:**
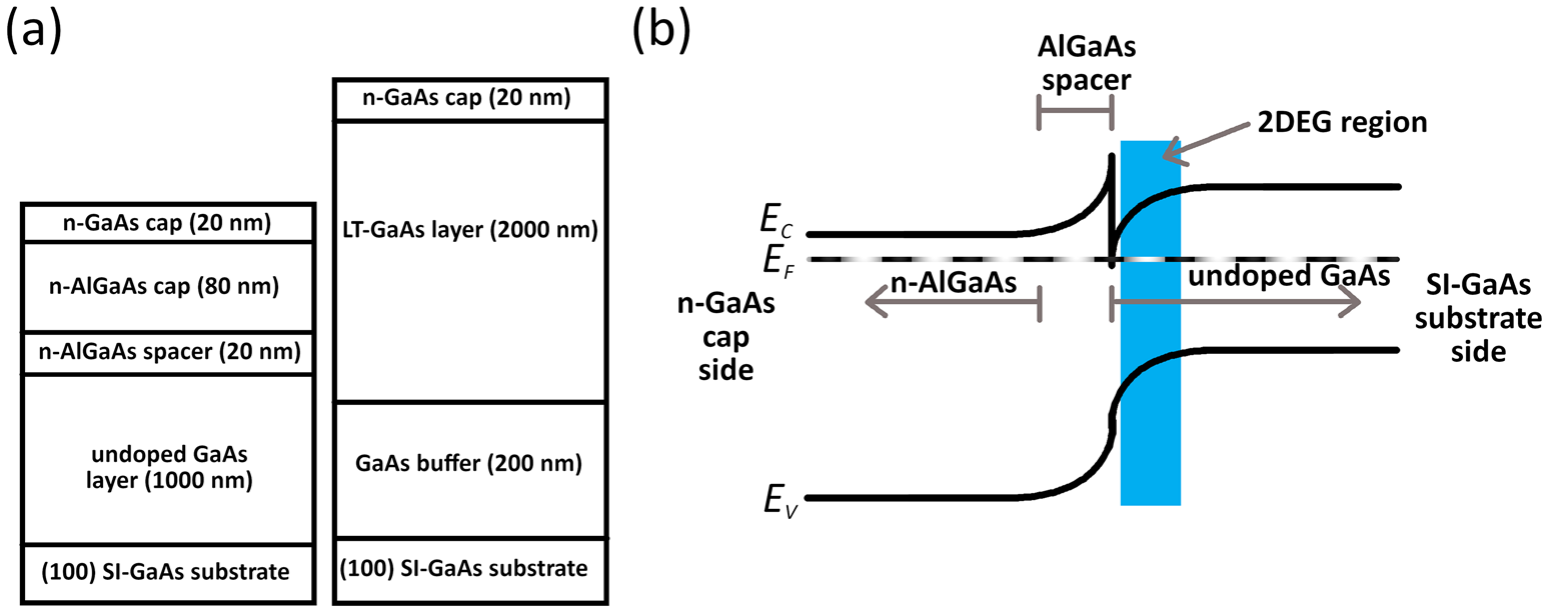
(**a**) Growth schematic of the n-AlGaAs/GaAs modulation-doped heterostructures and low-temperature grown GaAs and (**b**) band diagram of the n-AlGaAs/GaAs modulation-doped heterostructures. Figures are not to scale.

Figure 5a shows the growth schematics of the n-AlGaAs/GaAs MDH and LT-GaAs. The MDH layer was grown via a RIBER 32P molecular beam epitaxy on an epiready (100)-oriented SI-GaAs substrate. The substrate was first heated in situ at 590 °C for 10 min to remove its artificial oxides. The substrate temperature was then raised to 610 °C to facilitate the growth of a 1.5 µm GaAs buffer layer at a growth rate of 1 µm/hr. Afterward, this layer was followed by the growth of a 150 Å AlGaAs (x = 0.2) spacer at a growth rate of ~ 1.2 µm/hr. The silicon dopant effusion cell was then opened to facilitate the growth of an 800 Å n-AlGaAs donor layer by using the same growth conditions aside from a nominal doping concentration of ~ 1 × 10^17^ cm^−3^. The growth was then terminated through the growth of a 200 Å n-GaAs cap. The LT-GaAs layer was grown in the same MBE system, on a similar epiready (100) SI-GaAs substrate. The substrate was first heated in situ at 590 °C for 10 min to remove its artificial oxides. The substrate temperature was then raised to 630 °C for the growth of a 0.2 µm GaAs buffer. Afterward, the substrate temperature was lowered down to 270 °C where a 2 µm LT-GaAs thin film was grown. The substrate temperature was then raised to 600 °C in order to anneal the LTG-GaAs layer for 10 min. The growth then terminated through the growth of a 200 Å n-GaAs cap. All of the layers for this sample were grown at a growth rate of 1 µm/h.

Figure 5b shows the energy band diagram of a typical MDH. At the heterojunction between the highly doped n-GaAs layer and the undoped GaAs, a triangular quantum well is formed due to the alignment of the Fermi energy of the two materials. The MDH structure was originally designed to be used as a HEMT, as conduction electrons in the n-GaAs layer are designed to easily get trapped in the triangular quantum well (transistor channel), where they can move laterally at very low resistance (i.e. high mobility)^23,24^.

All of the samples used, namely two of MDH, LT-GaAs and SI-GaAs, underwent standard degreasing by immersion in trichloroethylene, acetone, and methanol. A MIDAS MDA-400 M mask aligner was used to transfer a dipole PCA structure with a gap $g$ = 5 µm onto the surfaces of the LT-GaAs, the SI-GaAs and one of the MDH wafers (to create MDH (Top)). The other MDH substrate (to create the MDH (Recessed) PCA) was also patterned using the same dipole PCA structure, albeit defocused, to obtain a slightly larger pattern than that of the original. This sample was etched in an acid piranha solution consisting of 1:8:80 volumetric ratio of H_2_SO_4_:H_2_O_2_:deionized H_2_O, which reached a depth of $d$ = 187 nm. After etching, the MDH (Recessed) substrate was patterned with the same dipole PCA pattern for metallization. AuGe/Ni/Au with nominal thicknesses of 55/15/85 nm were evaporated onto the samples by resistive evaporation and electron beam deposition. After metal lift-off, all of the PCAs were annealed inside a tube furnace at 400 °C under nitrogen gas-rich environment for 1 min.

The THz emission characteristics of the samples were measured using a standard THz-TDS spectroscopy setup. The 780 nm line of a Menlo C-fiber femtosecond fiber laser with pulse duration of 100 fs pulse duration and 100 MHz repetition rate was used. The laser beam was split into pump and probe beams using a beam splitter. The pump beam was used to excite the emitter samples and the probe beams was used to optically gate a commercial 3.4 µm LT-GaAs dipole detector. The pump and probe powers were both maintained at 9.5 mW, unless otherwise stated. The PCA emitters were biased with a 32 V peak to peak square wave at a frequency of 20 kHz.
